# Supplementary figures and images for: Development of a mouse model of ascending infection and preterm birth
Source: PLoS One. 2021 Dec 2;16(12):e0260370. doi: 10.1371/journal.pone.0260370 (PMC8638907; doi:10.1371/journal.pone.0260370)

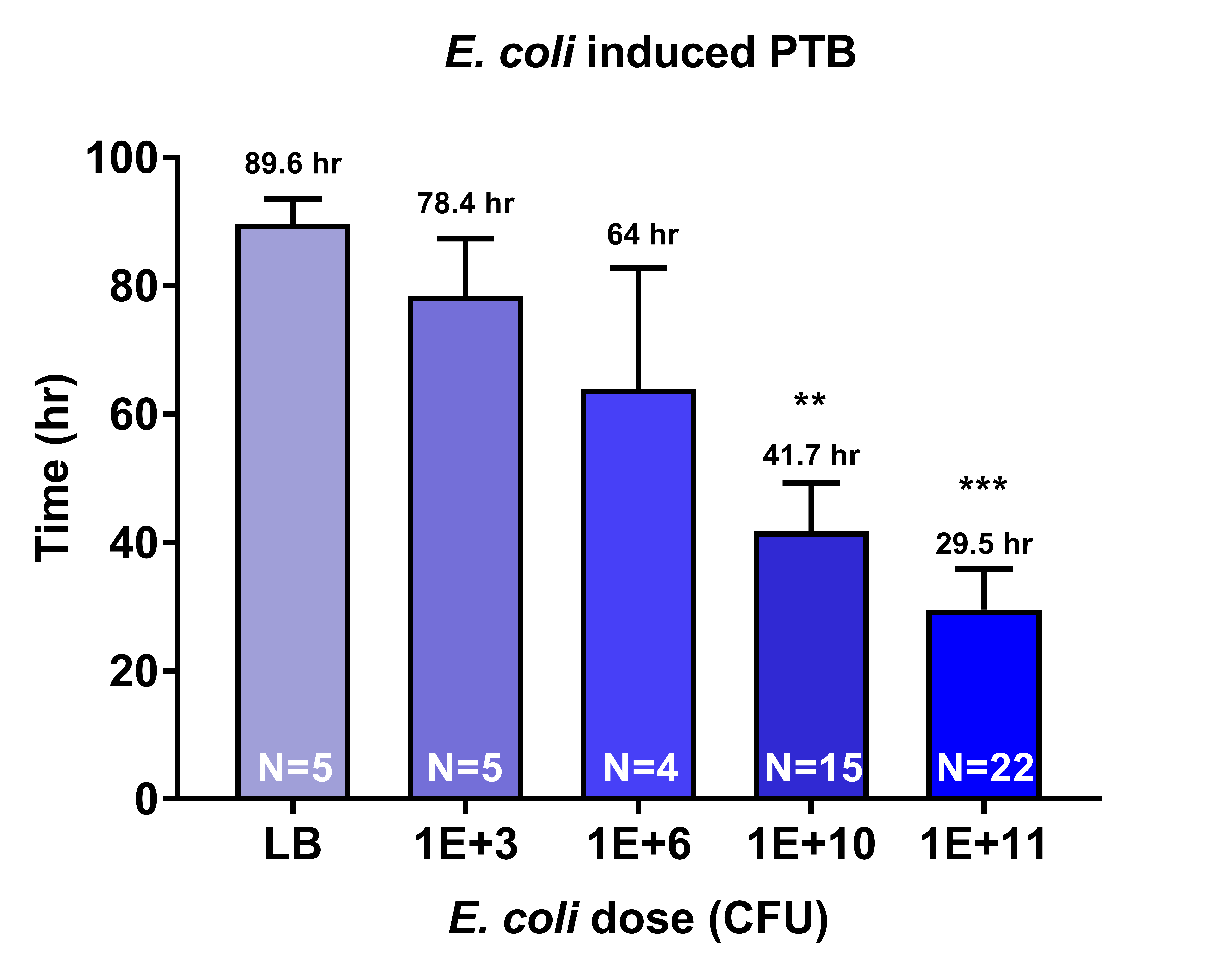

Supplement: S1 Fig — Higher doses of E. coli (1011 CFU and 1010 CFU) delivered significantly shorter time frame compared to control (LB) (P<0.001 and P = 0.002, respectively). Low dose of E. coli (106 CFU and 103 CFU) delivered in shorter time frame compared to control, however not significantly (P = 0.17 and P = 0.3, respectively). CFU-colony forming unit. (TIF) [file pone.0260370.s001.tif]

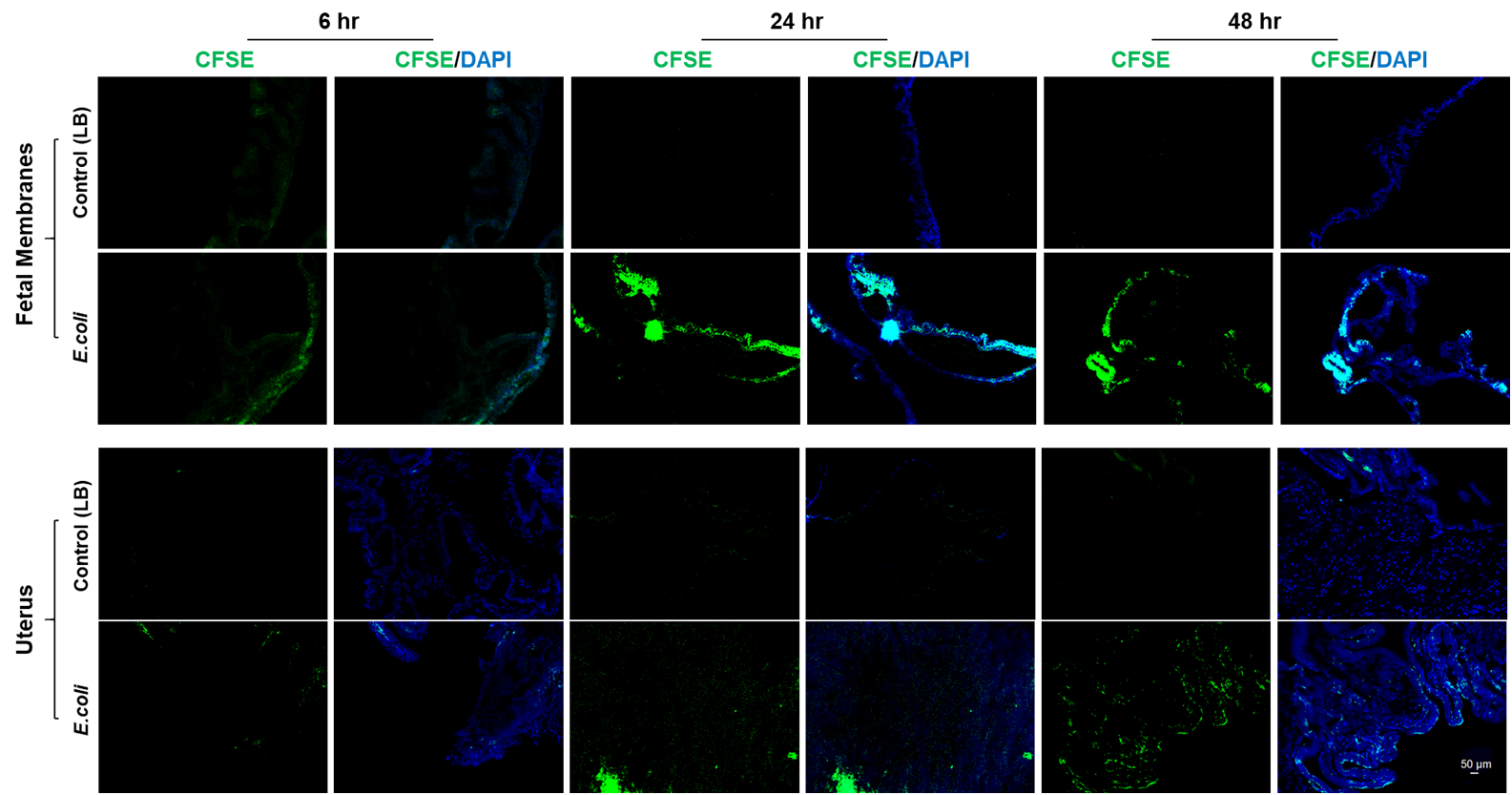

Supplement: S2 Fig — Scale bar, 50 μm. (TIF) [file pone.0260370.s002.tif]

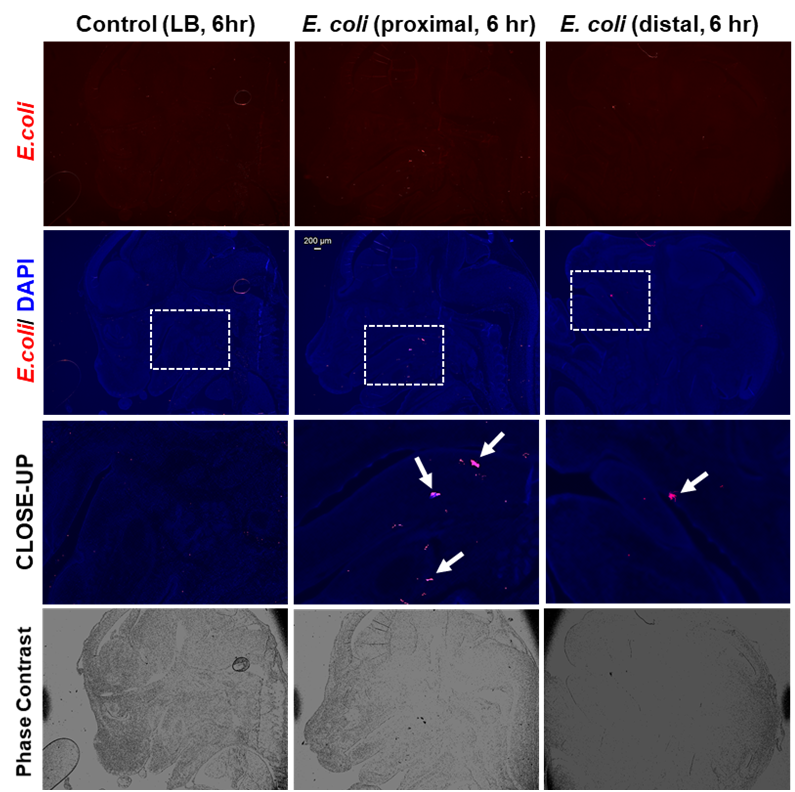

Supplement: S3 Fig — Scale bar, 200 μm. (TIF) [file pone.0260370.s003.tif]

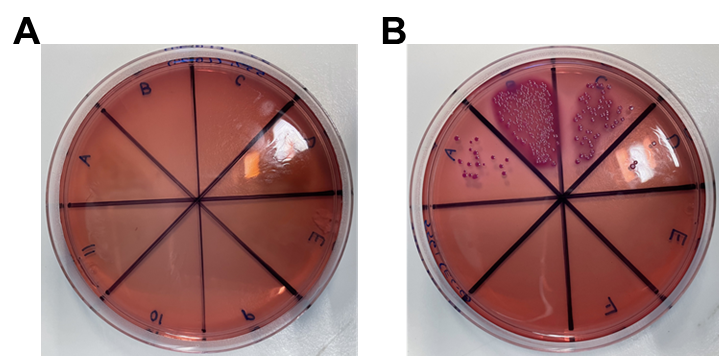

Supplement: S4 Fig — Representative images of amniotic fluid recovered bacterial growth on MacConkey agar. (A) Control plate. Negative for E. coli growth. (B) Positive E. coli growth plate. (TIF) [file pone.0260370.s004.tif]

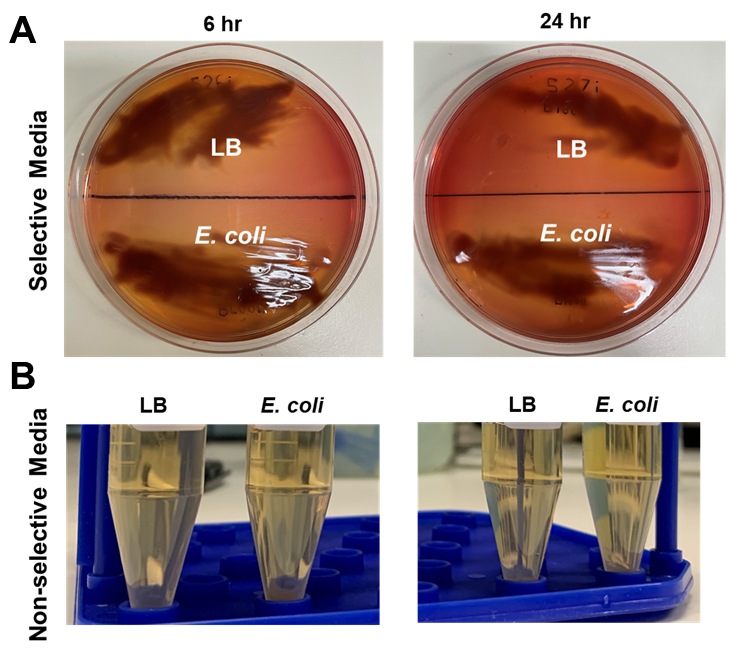

Supplement: S5 Fig — (A) MacConkey’s agar culture showed no bacterial growth (N = 3). (B) Confirmational culture in the liquid nutritional broth using the samples directly transferred from MacConkey’s agar culture from A. (TIF) [file pone.0260370.s005.tif]

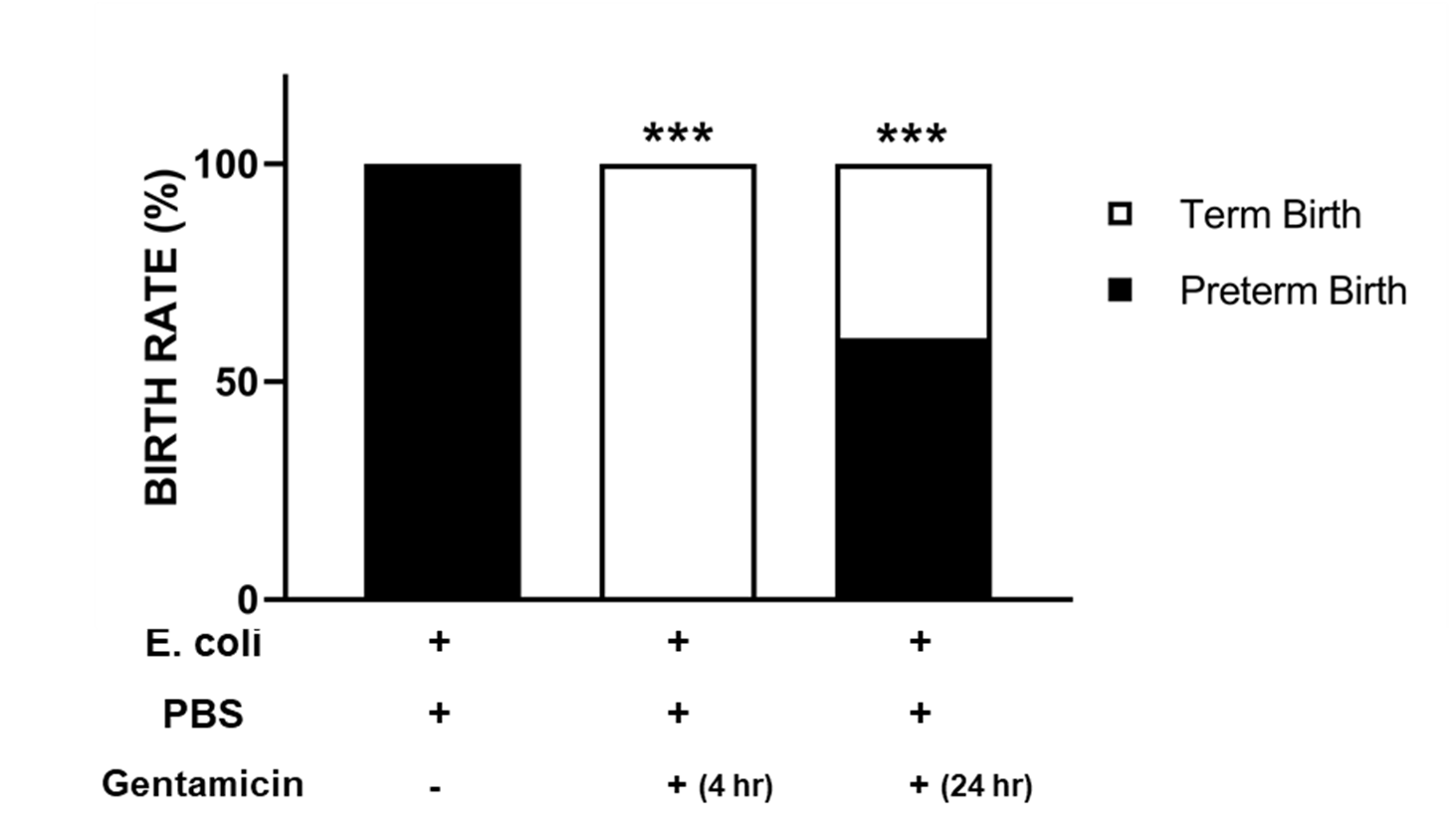

Supplement: S6 Fig — Gentamicin (20 mg/kg) at 4 hr after E. coli administration showed 100% prolonged gestation to term delivery compared to controls (PBS only) (P< 0.001) (N = 3); however, same dose given after 24 hours shows 40% effect on increased length of gestation to term delivery compared to controls (P< 0.001) (N = 5). (TIF) [file pone.0260370.s006.tif]
